# Polycystin‐1 Controls Cell Cycle Kinetics, Cell Cycle Exit, and Differentiation of Neural Progenitor Cells

**DOI:** 10.1096/fj.202503816R

**Published:** 2026-04-25

**Authors:** Natalie Winokurow, Stefan Schumacher

**Affiliations:** ^1^ Institute of Molecular and Cellular Anatomy Ulm University Ulm Germany

**Keywords:** autosomal dominant polycystic kidney disease (ADPKD), cell cycle, neurogenesis, polycystin‐1, polycystin‐2, progenitor cells

## Abstract

In neocortical neurogenesis, neural progenitor cells (NPCs) give rise to diverse types of neurons. During this process, the balance between proliferation and differentiation, cell division mode determination, and cell fate specification are all intimately linked to the cell cycle of the NPCs. The cell cycle length hypothesis states that G1‐phase lengthening switches NPCs from proliferative to neurogenic divisions. Meanwhile, however, the importance of S‐phase shortening in differentiating NPCs emerges. Mutations in the polycystin‐1 (PC1)‐ and polycystin‐2 (PC2)‐encoding genes are causative for the development of autosomal dominant polycystic kidney disease (ADPKD), a prominent feature of which is unbalanced cell proliferation. Here, we examine the impact of PC1 and PC2 on cell cycle kinetics, cell cycle exit, and the neuronal differentiation of NPCs. Loss‐of‐function analysis and cell‐based assays demonstrate that NPCs with reduced PC1 expression exhibit a longer cell cycle with an increased S‐phase duration. The cell cycle exit and the neuronal differentiation of these cells are significantly delayed. A strong tendency towards similar phenotypes is observed when reducing PC2 expression in NPCs. Thus, decreasing PC1 expression expands the pool of slowly cycling NPCs. These results highlight the significance of S‐phase shortening in neurogenesis and may contribute to a better understanding of ADPKD pathophysiology.

## Graphical abstract

Earlier in neurogenesis, *knockdown* of polycystin‐1 (PC1) expression increases the cell cycle length of neural progenitor cells (NPCs) by extending the S‐phase and probably lengthening the G1‐phase. Subsequently, reduced PC1 expression causes delayed cell cycle exit and delayed neuronal differentiation of NPCs. Thus, by controlling cell cycle kinetics and cell cycle exit, PC1 adjusts the size of the important pool of slowly cycling NPCs and connects S‐phase shortening with neurogenesis.

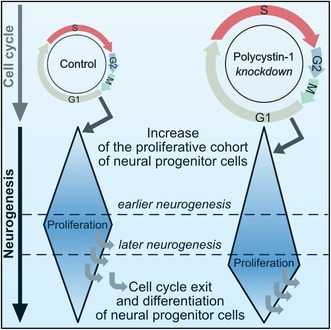

## 1 Introduction

Mutations in the genes encoding polycystin‐1 (PC1) and polycystin‐2 (PC2) account for almost all cases of autosomal dominant polycystic kidney disease (ADPKD), characterized by progressive cyst formation and enlargement [1]. Unbalanced cell proliferation contributes to the cystic phenotype [2, 3, 4]. Recent data show that PC1 and PC2 are expressed in mouse embryonic neocortical neural progenitor cells (NPCs). Reducing PC1 or PC2 expression in NPCs causes more symmetric, proliferative cell divisions [5]. While the cell cycle length hypothesis states that G1‐phase lengthening is key to switching NPCs from proliferative to neurogenic cell divisions [6, 7, 8], the relevance of S‐phase shortening is emerging [9, 10, 11]. Yet how PC1 and PC2 impact cell cycle‐coordinated NPC development is unexplored.

## 2 Methods

### 2.1 Cell Culture

NPCs were prepared from the neocortical hemispheres of C57BL/6J mouse embryos at embryonic day 13.5 (E13.5) as described [5]. The cells were cultured in Neurobasal A medium supplemented with B‐27, 0.5 mM L‐glutamine and 100 U/mL penicillin/streptomycin. To promote NPC proliferation, 10 ng/mL recombinant basic fibroblast growth factor (Thermo Fisher Scientific #100‐18B‐50UG) was added. The NPCs were transfected with Lipofectamine Stem transfection reagent (Thermo Fisher Scientific #STEM00003) 1 day after plating (days in vitro 1; DIV1).

### 2.2 DNA Constructs

The shRNA constructs Pkd1‐kd and Pkd2‐kd have been validated and characterized in a recent study [5]. The construct M‐PKD2 (OF2‐3), here designated MYC‐PC2, was a gift from Gregory Germino (Addgene #21370) [12].

### 2.3 Immunocytochemistry

Indirect co‐immunostainings were conducted as described [5] using fluorescence‐labeled secondary antibodies (Alexa Fluor 488, 568, 647). Primary antibodies applied: monoclonal rat anti‐BrdU (clone BU1/75), mouse anti‐IdU/BrdU (clone B44), mouse anti‐BrdU (clone BU‐33), rabbit anti‐Ki‐67 (clone D3B5), mouse anti‐MAP2 (clone HM‐2), and rabbit or chick polyclonal antibodies to MYC‐tag (Cell Signaling #2272) or GFP (in‐house), respectively. For BrdU and IdU stainings, an antigen retrieval step with HCl was performed.

### 2.4 Image Acquisition and Data Analysis

Image acquisition and data analysis were conducted as described [5] by investigators blinded to the experimental conditions. IdU and BrdU labelings (5 μg/mL each) were performed for the indicated periods. Triple‐immunostained cells (GFP/BrdU/IdU + BrdU) were analyzed by means of confocal images (20× objective, Leica HCX PL APO CS 20.0 × 0.7; 2.3 × digital zoom). *Z*‐stacks were collected at 0.8‐μm steps. For the cumulative BrdU labeling approach, the number of BrdU‐positive transfected NPCs was counted for each optical field of view (epifluorescence microscopy; 40× objective, Leica HCX PL APO 40.0 × 0.75 PH2). For cell cycle exit and differentiation analysis, triple‐immunostained cells (GFP/BrdU/Ki‐67 or GFP/BrdU/MAP2, respectively) were analyzed by means of confocal images (40× objective, Leica HCX PL APO CS 40.0 × 1.2 oil). *Z*‐stacks were collected at 1.0 μm steps.

The S‐phase length was calculated either by the IdU/BrdU double labeling [13] or by the cumulative BrdU labeling approach [14]. The latter was employed to determine the total cell cycle length.

### 2.5 Statistical Analysis

Statistical analysis was performed using Prism10 (GraphPad) software. For each analysis, three biological replicates were conducted. For all replicates, independent NPC cell cultures were established. The cell suspension was split in order to be transfected with the different constructs (e.g., kdcontrol, Pkd1‐kd, Pkd2‐kd). Because of this matching, the significance between groups (means of the three biological replicates, aggregated from all data points according to the scatter plots) was assessed by the Friedman test followed by post hoc analysis (Dunn's multiple comparison test). Values of *p* < 0.05 were considered significant. The data points of the scatter plots were derived from the analysis of confocal images or optical fields of view.

All statistical data are presented in Table S1.

## 3 Result

### 3.1 PC1 Regulates the Cell Cycle and the S‐Phase Length of NPCs

**FIGURE 1:**
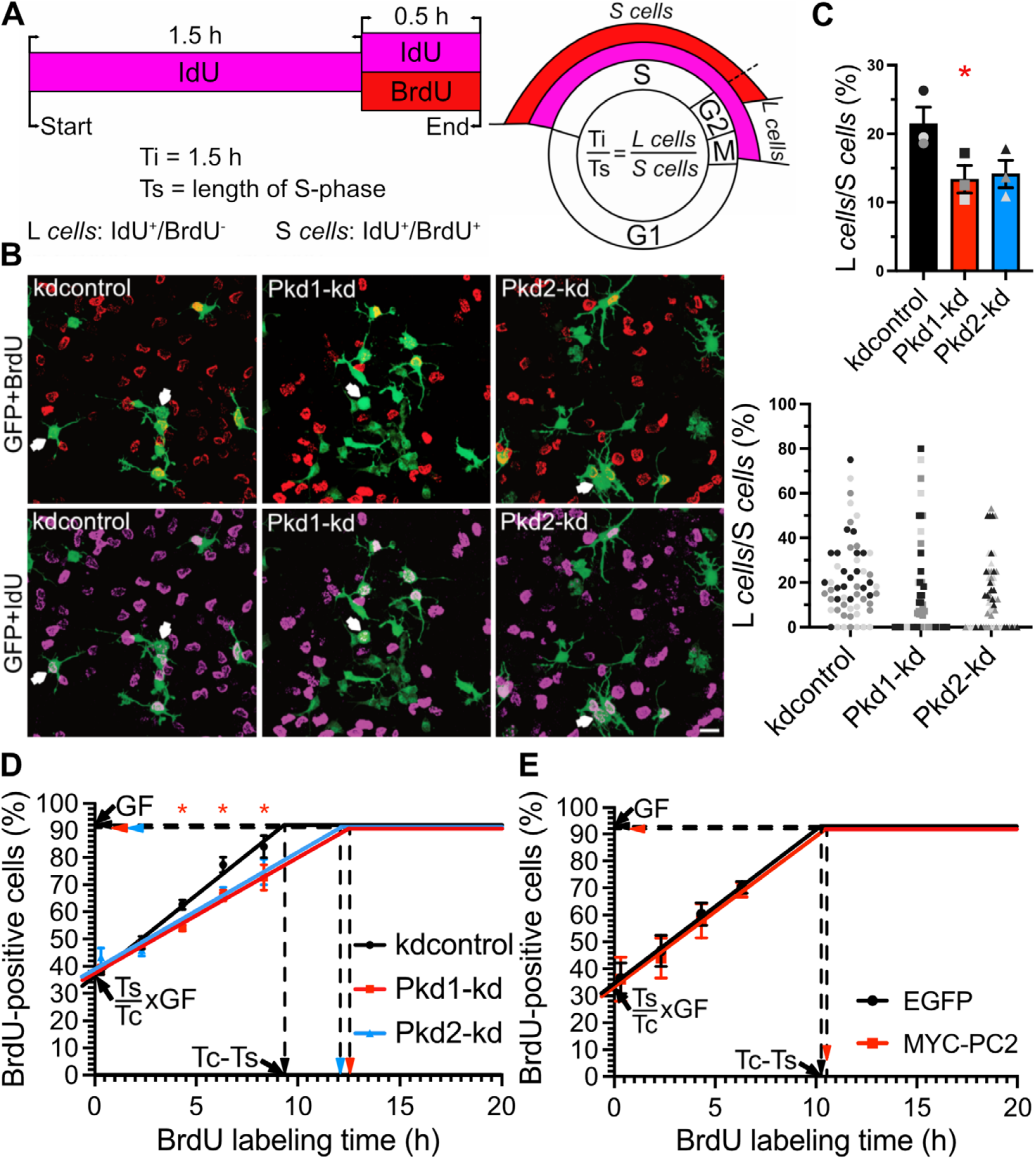
Reduced PC1 expression significantly extends the S‐phase and lengthens the cell cycle of NPCs. (A) Schema of the time schedule and calculation of the cell cycle kinetics by IdU/BrdU double labeling. During Ti, the interval when NPCs are exposed to IdU (magenta) but not BrdU (red), a fraction of these cells leave S‐phase (L *cells*, IdU^+^/BrdU^−^). IdU^+^/BrdU^+^‐NPCs belong to the S‐phase cohort (S *cells*) at the end of the experiment. The ratio between the number of L c*ells* and S *cells* equals the ratio between Ti and Ts (length of S‐phase). (B) Representative images of NPCs transfected as indicated, and stained for GFP (green, to indicate transfected cells), BrdU (red), and IdU (magenta). White arrows highlight NPCs that were labeled by IdU but not BrdU. Scale bar, 15 μm. (C) Ratio of the number of L *cells* to the number of S *cells* was determined. Data are presented as mean ± SEM (*p < 0.05). The means of each replicate are indicated in the diagram (gray tone‐coded for each biological replicate). The scatter plot illustrates the distribution of the data, gray tone‐coded for each biological replicate. One dot represents the L *cells* to S c*ells* ratio of the transfected NPCs of one analyzed confocal image. (D, E) Cumulative BrdU labeling curves. The BrdU labeling started on DIV3 and was completed 44 h later. Dashed horizontal or vertical lines indicate the growth fraction (GF) or the time point at which the BrdU labeling reaches a plateau (Tc‐Ts), respectively. Data are presented as mean ± SEM (*p < 0.05).

We examined the S‐phase length (Ts) of NPCs with reduced PC1 or PC2 expression. To calculate Ts, proliferating NPCs were sequentially exposed to IdU, followed by IdU/BrdU double labeling (Figure 1A) [13]. We determined the ratio of L *cells*, which exited S‐phase during IdU labeling, to S *cells* staying in S‐phase during IdU/BrdU double labeling. According to the equation Ti/Ts = L *cells*/S *cells* (Figure 1A), Ts was calculated: 7.0 h for kdcontrol NPCs, and 11.2 h or 10.6 h for Pkd1‐kd (PC1 *knockdown*) or Pkd2‐kd (PC2 *knockdown*) cells, respectively. While the L *cells*/S *cells* ratio was significantly reduced by Pkd1‐kd, the effect of Pkd2‐kd on this ratio was statistically not significant (Figure 1A–C).

To confirm the S‐phase lengthening by PC1 or possibly PC2 *knockdown*, we performed cumulative BrdU labeling of proliferating NPCs (Figure 1D) [14]. First, the percentage of BrdU‐positive cells at the plateau of the respective BrdU labeling curve, indicating the growth fraction (GF), was assessed. Next, the duration of the total cell cycle excluding the S‐phase length (Tc‐Ts, corresponding to the x‐axis intercept) was deduced. This interval (G2/M/G1) was longer in PC1 or PC2 *knockdown* NPCs. Then, “Ts/Tc X GF”, corresponding to the Y‐axis intercept, was determined (Figure 1D). Subsequent calculations [14] yielded a Tc and Ts prolongation of PC1 and PC2 *knockdown* NPCs. Specifically, Ts was 6 h (Tc = 15.4 h) for kdcontrol NPCs, and 8.8 h (Tc = 21.4 h) or 8.7 h (Tc = 20.8 h) for Pkd1‐kd or Pkd2‐kd cells, respectively. However, here again, the effect of Pkd2‐kd was statistically not significant.

An envisaged PC1 gain‐of‐function was unsuccessful, because only a few healthy NPCs expressed PC1, precluding any analysis. Although a fraction of NPCs expressing a MYC‐PC2 construct exhibited a poor tolerance to BrdU incorporation over extended periods, a reliable cumulative BrdU labeling curve was obtained. MYC‐PC2 (Ts = 5.9 h; Tc = 16.5 h) neither changed Ts nor Tc when compared to EGFP (Ts = 6.0 h; Tc = 16.3 h) overexpression (Figure 1E).

In summary, reducing PC1 expression lengthens the cell cycle of NPCs and increases their S‐phase duration.

### 3.2 Knockdown of PC1 Expression Affects Cell Cycle Exit and the Neuronal Differentiation of NPCs

**FIGURE 2:**
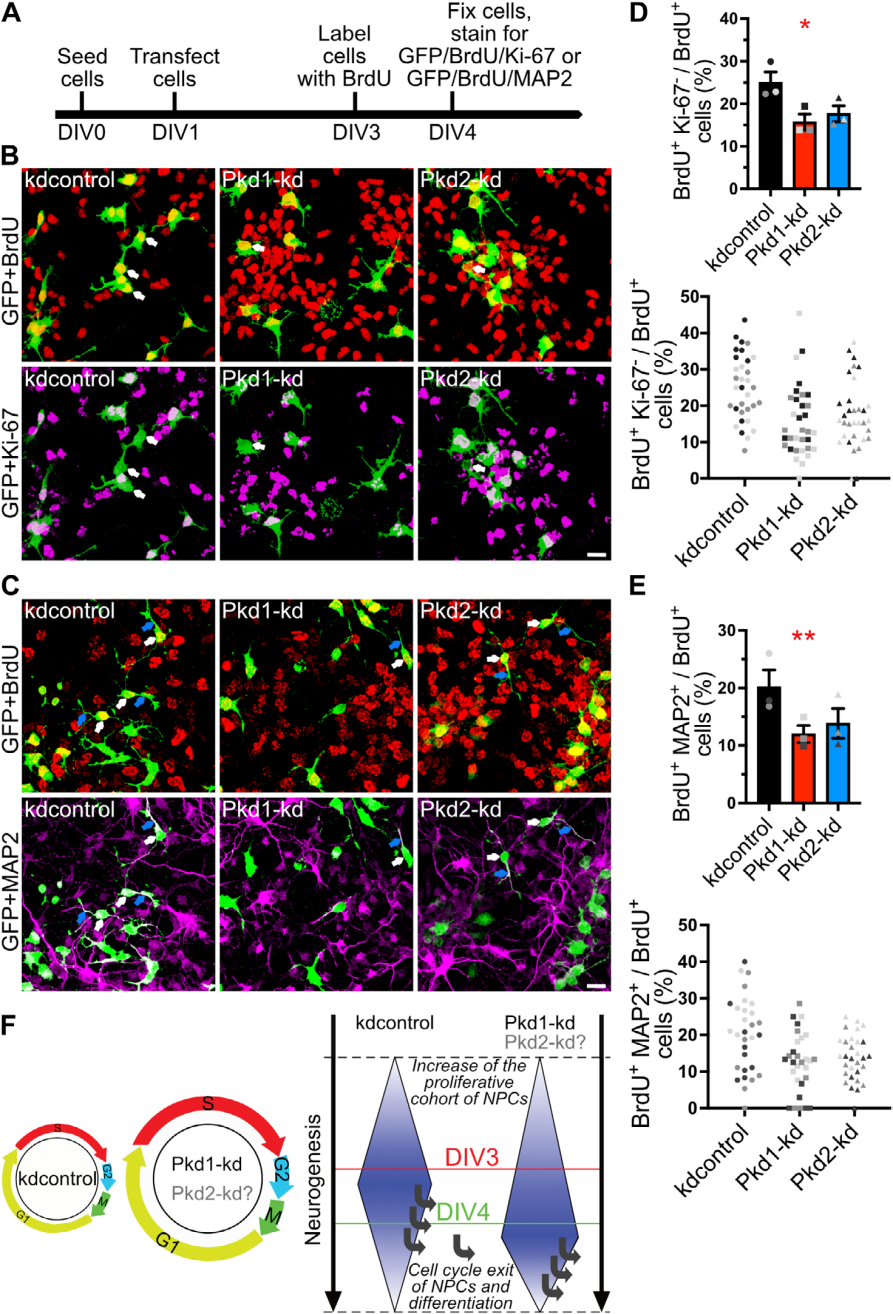
Knockdown of PC1 expression significantly delays the cell cycle exit and neuronal differentiation of NPCs. (A) Time schedule of the experiment; DIV, days in vitro. (B) Representative images of NPCs transfected as indicated, and stained for GFP (green, to indicate transfected cells), BrdU (red), and Ki‐67 (magenta). White arrows highlight transfected NPCs that left the cell cycle 24 h after BrdU labeling (BrdU^+^/Ki67^−^). (C) Representative images of NPCs transfected as indicated, and stained for GFP (green, to indicate transfected cells), BrdU (red), and MAP2 (magenta). White arrows highlight transfected NPCs that were labeled by the neuronal marker MAP2 24 h after BrdU labeling (BrdU^+^/MAP2^+^). Scale bar, 15 μm (B, C). (D, E) Percentages of BrdU^+^/Ki67^−^ (D) or BrdU^+^/MAP2^+^ (E) transfected cells over the total number of BrdU^+^ transfected cells were determined. Data are presented as mean ± SEM (*p < 0.05, **p < 0.01). A scatter plot illustrates the distribution of the data. One dot represents the percentages of BrdU^+^/Ki67^−^ (D) or BrdU^+^/MAP2^+^ (E) transfected cells, respectively, of one analyzed confocal image. (F) Schematic summary of the results. Silencing PC1 expression prolongs the cell cycle of NPCs by extending the S‐phase and probably the G1‐phase, delays the cell cycle exit, and delays the differentiation of NPCs. This way, PC1 *knockdown* decreases the proliferative cohort of NPCs early in cell culture (DIV3) and restrains neuronal differentiation later (DIV4). Although causing similar phenotypes, the effects of PC2 *knockdown* were statistically not significant. This limitation is indicated by displaying “Pkd2‐kd” in light gray with a question mark.

We wondered whether reduced PC1 or PC2 expression would delay the cell cycle exit and the differentiation of NPCs. To examine this hypothesis, the percentages of either BrdU^+^/Ki‐67^−^ transfected NPCs (Figure 2A,B,D; Figure S1) or BrdU^+^/MAP2^+^ transfected NPCs (Figure 2A,C,E; Figure S1), respectively, were assessed 24 h after BrdU labeling. Actually, the number of transfected NPCs leaving the cell cycle (BrdU^+^/Ki‐67^−^) and the number of NPCs differentiating to neurons (BrdU^+^/MAP2^+^) significantly decreased by PC1 *knockdown*. PC2 *knockdown* resulted in similar trends without reaching statistical significance. As PC1 *knockdown* does not significantly alter the number of apoptotic NPCs [5], we conclude that reducing PC1 expression delays the cell cycle withdrawal and the differentiation of NPCs.

## 4 Discussion

Reducing PC1 expression in NPCs (1) prolongs the cell cycle by extending the S‐phase, (2) delays cell cycle exit and the differentiation of NPCs. We conclude that PC1 promotes the differentiation of NPCs (i) by shortening Tc and Ts, (ii) by accelerating the cell cycle exit (Figure 2F). We suggest that PC1 and PC2 are functionally interlinked as PC2 overexpression alone has no impact on Tc and Ts, and *knockdown* of PC1 or PC2 affects NPCs similarly (this study and [5], and also see “Limitations”).

PC1 *knockdown* also causes an increase of the G2/M/G1 length (Tc‐Ts). Assuming that PC1 *knockdown* did not dramatically elongate the G2/M‐phase (our preliminary data suggest a similar G2/M length for all conditions; data not shown), one would expect that also the G1‐phase (T_G1_) lengthens (Figures 1D and 2F). Thus, PC1 *knockdown* associates NPCs with self‐renewal divisions (longer S‐phase), but additionally appears to enable them to integrate differentiation signals (longer G1‐phase). Exciting work has identified NPCs in the neocortical ventricular zone fate‐restricted for upper‐layer neurogenesis at times of earlier lower‐layer neurogenesis [15, 16]. These NPCs remain in the cell cycle longer for slowly expanding the progenitor pool. Silencing PC1 expression might contribute to a similar progenitor cell type.

Li et al. elaborated the helix–loop–helix family member Id2 in connecting PC1 and PC2 to cell cycle regulation in kidney epithelial cells by controlling nuclear p21^CIP1^ expression and S‐phase entry [3]. However, reducing PC1 (and also PC2) expression here caused a longer S‐phase but a shorter G1‐phase.

Mechanistically, Ts depends on the speed of the replication fork [17]. In the erythroid lineage, a switch from self‐renewal to maturational cell divisions depends on Ts shortening. However, not only Ts shortens during this switch, but also Tc and T_G1_, meaning that in self‐renewal divisions Ts, Tc, and T_G1_ lengthen [17]. The cyclin‐dependent kinase inhibitor p57^KIP2^, known to control G1/S transition [18], was found in self‐renewing erythroid progenitors to prolong Ts by slowing the replication fork speed [17]. Interestingly, p57^KIP2^ was functionally linked to the cell cycle slowing down in a subset of embryonic ventricular zone NPCs destined to become adult neural stem cells [19]. Therefore, p57^KIP2^ might be a promising candidate to mechanistically link PC1 with G1‐ and S‐phase regulation coupled to cell fate decisions.

In ADPKD, cyst formation does not require a complete loss of polycystin function [1]. Thus, reduced PC1 expression might cause kidney epithelial cells to revert to a progenitor stage, more error‐prone for the tasks of differentiated cells.

## 5 Limitations

Because the NPCs were grown in cell culture, our results stress the relevance of cell‐intrinsic cues which guide neurogenesis [20, 21]. Limitations of this in vitro approach are the missing three‐dimensional tissue architecture and lamination‐directed signaling. In vivo validation of our findings could be performed in the embryonic neocortex (e.g., by in utero *electroporation*), or by employing organoid models. A second limitation is related to the PC2 *knockdown* results. Although causing similar phenotypes, the effects of PC2 *knockdown*, in contrast to PC1 *knockdown*, were statistically not significant. A conceivable technical reason to explain this mismatch could be a weaker *knockdown* capacity of Pkd2‐kd. Physiologically, it may be possible that PC2 cannot compensate for PC1 silencing, but PC1 could—to some extent—offset the reduction of PC2 function. Lastly, we cannot exclude the possibility that PC2, in contrast to PC1, does not affect the cell cycle substantially.

## Author Contributions

S.S. conceived the project. N.W. and S.S. designed and performed the experiments. N.W. and S.S. analyzed the data. N.W. and S.S. wrote the manuscript.

## Funding

The authors have nothing to report.

## Conflicts of Interest

The authors declare no conflicts of interest.

## Supporting information


**Figure S1:** fsb271844‐sup‐0001‐FigureS1.pdf.

**Table S1:** fsb271844‐sup‐0002‐TableS1.pdf.

## Data Availability

Data will be made available on request.
