# Supplementary material for: Polycystin‐1 Controls Cell Cycle Kinetics, Cell Cycle Exit, and Differentiation of Neural Progenitor Cells
Source: FASEB J. 2026 Apr 25;40:e71844. doi: 10.1096/fj.202503816R (PMC13109810; doi:10.1096/fj.202503816R)
Supplement: Supplementary file 1 — Figure S1: fsb271844‐sup‐0001‐FigureS1.pdf. [file FSB2-40-e71844-s002.pdf]

Supporting Information

Supporting Information Figure S1

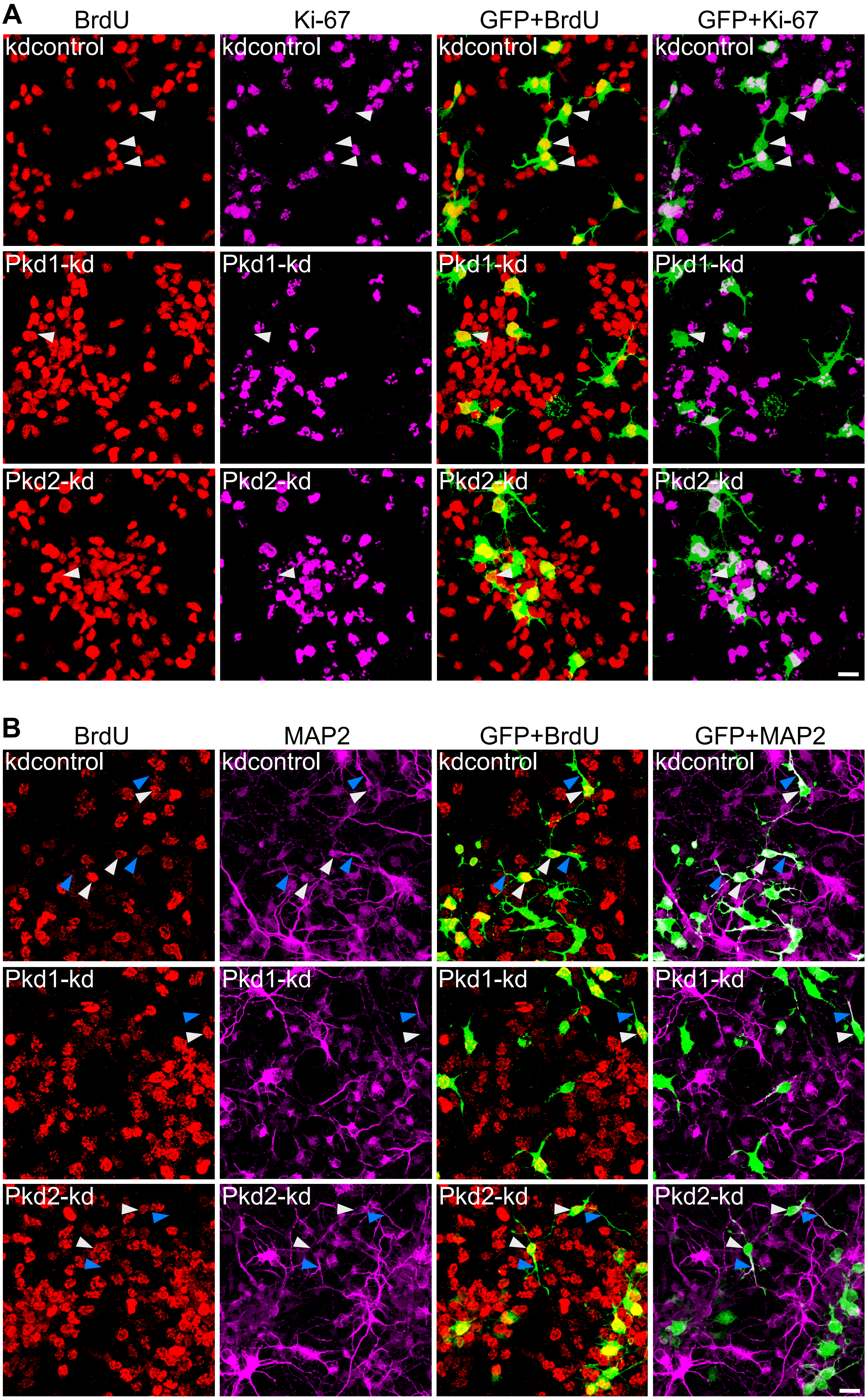

Figure S1. Knockdown of PC1 expression delays the cell cycle exit and the neuronal differentiation of NPCs. Related to Figure 2.

(A) Representative images of NPCs transfected as indicated, and stained for GFP (green, to indicate transfected cells), BrdU (red), and Ki-67 (magenta). White arrows highlight transfected NPCs that left the cell cycle 24 h after BrdU labeling ($BrdU^+/Ki67^-$).

(B) Representative images of NPCs transfected as indicated, and stained for GFP (green, to indicate transfected cells), BrdU (red), and MAP2 (magenta). White arrows highlight transfected NPCs that were labeled by the neuronal marker MAP2 24 h after BrdU labeling ($BrdU^+/MAP2^+$).Scale bars, 15 μm (A, B).
