# Supplementary material for: Polycystin‐1 Controls Cell Cycle Kinetics, Cell Cycle Exit, and Differentiation of Neural Progenitor Cells
Source: FASEB J. 2026 Apr 25;40:e71844. doi: 10.1096/fj.202503816R (PMC13109810; doi:10.1096/fj.202503816R)
Supplement: Supplementary file 2 — Table S1: fsb271844‐sup‐0002‐TableS1.pdf. [file FSB2-40-e71844-s001.pdf]

Supporting Information Table 1

Number of replicates for each experiment, the number of surveyed cells, the statistics including mean ± SEM for each group, and the related statistical tests.

Figure	Experimental schedule<br>Constructs applied	Number of independent experiments (biological replicates)	Number of data values measured (number of cells analyzed,<br>number of WB bands analyzed)	Parameter analyzed<br>Constructs: mean ± SEM	Statistical test
1C	Transfection of cortical NPCs with the following constructs on DIV1:<br>1) kdcontrol<br>2) Pkd1-kd<br>3) Pkd2-kd<br>Two days after transfection, the cells were consecutively labeled by IdU alone (1.5 h) followed by IdU/BrdU double-labeling (0.5 h), fixed, and processed for immunocytochemistry. After triple-labeling (GFP, BrdU, IdU + BrdU), the number of IdU^{+}/BrdU^{-}-NPCs (L cells), and the number of IdU^{+}/BrdU^{+}-NPCs (S cells) were determined; finally, the ratio of IdU^{+}/BrdU^{-} (L cells)- to IdU^{+}/BrdU^{+} (S cells)-cells for each experimental condition was deduced.	3	At least 1,200 transfected NPCs collected from 57 confocal images were analyzed for each experimental condition (19 images per replicate).	Ratio of L cells/ S cells (%)<br>1) 21.5 ± 2.4<br>2) 13.4 ± 2.0<br>3) 14.1 ± 2.0	The means of the 3 biological replicates were statistically analyzed. Friedman test followed by post-hoc multiple comparison analysis (Dunn).
1D	Transfection of cortical NPCs with the following constructs on DIV1:<br>1) kdcontrol<br>2) Pkd1-kd<br>3) Pkd2-kd<br>The NPCs continuously got BrdU (5 µg/ml) starting two days after transfection (DIV3). Fractions of cells (2 coverslips per experiment for each experimental condition) were fixed 0.3 h, 2.3 h, 4.3 h, 6.3 h, 8.3 h, and 44 h after the start of the BrdU labeling. From these data, the cumulative BrdU-labeling curve was generated.	3	At least 200 transfected NPCs collected from 15 visual fields (5 per replicate) were analyzed for each experimental condition and each point of time.	BrdU-positive transfected cells (%)<br>Time zero (0.3 h):<br>1) 38.0 ± 1.1<br>2) 38.7 ± 1.8<br>3) 43.3 ± 3.3<br>Time 2,3 h:<br>1) 48.7 ± 2.4<br>2) 48.0 ± 1.2<br>3) 46.0 ± 2.3<br>Time 4.3 h:<br>1) 62.7 ± 1.8<br>2) 54.0 ± 1.2	The means of the 3 biological replicates were statistically analyzed. Friedman test followed by post-hoc multiple comparison analysis (Dunn).

				3) 55.3 ± 1.8<br><br>Time 6.3 h:<br>1) 77.3 ± 2.9<br>2) 66.0 ± 2.0<br>3) 68.0 ± 1.2<br><br>Time 8.3 h:<br>1) 84.0 ± 4.2<br>2) 72.7 ± 4.7<br>3) 74.7 ± 4.7<br><br>Time 44 h:<br>1) 92.0 ± 2.3<br>2) 91.3 ± 1.8<br>3) 90.7 ± 0.7	
1E	Transfection of cortical NPCs with the following constructs on DIV1:<br>1) EGFP<br>2) MYC-PC2<br>The NPCs continuously got BrdU (5 μg/ml) starting two days after transfection (DIV3). Fractions of cells (2 coverslips per experiment for each experimental condition) were fixed 0.3 h, 2.3 h, 4.3 h, 6.3 h, 8.3 h, and 44 h after the start of the BrdU labeling. From these data, the cumulative BrdU-labeling curve was generated.	3	At least 300 transfected NPCs collected from at least 36 visual fields (12 per replicate) were analyzed for each experimental condition and times 0.3 h, 2.3 h, 4.3 h, and 6.3 h. Due to problems of cell viability in the case of MYC-PC2 overexpression (poor tolerance to BrdU incorporation over extended times), a reliable data point at time 8.3 h could not be obtained. Due to the problematic of cell viability, at least 120 transfected NPCs collected from at least 12 visual fields (4 per replicate) were analyzed for the time 44 h.	BrdU-positive transfected cells (%)<br><br>Time zero (0.3 h):<br>1) 36.5 ± 5.6<br>2) 36.2 ± 8.2<br><br>Time 2.3 h:<br>1) 46.7 ± 5.8<br>2) 44.1 ± 7.5<br><br>Time 4.3 h:<br>1) 60.4 ± 4.2<br>2) 57.9 ± 6.3<br><br>Time 6.3 h:<br>1) 70.3 ± 2.2<br>2) 69.4 ± 2.7<br><br>Time 44 h:<br>1) 93.0 ± 0.5<br>2) 91.6 ± 1.3	The means of the 3 biological replicates were statistically analyzed. Friedman test followed by post-hoc multiple comparison analysis (Dunn).

2D	Transfection of cortical NPCs with the following constructs on DIV1:<br>1) kdcontrol<br>2) Pkd1-kd<br>3) Pkd2-kd<br>The cells were BrdU-labeled on DIV3 for three hours, fixed 24 h later on DIV4, and subjected to immunocytochemistry. The percentage of $BrdU^+$/$Ki\text{-}67^-$ NPCs (NPCs that left the cell cycle after BrdU labeling) for each experimental condition was determined.	3	At least 900 transfected NPCs collected from 33 confocal images (11 per replicate) were analyzed for each experimental condition.	$BrdU^+$ $Ki\text{-}67^-$ / $BrdU^+$ cells (%)<br><br>1) 25.0 ± 2.5<br>2) 15.7 ± 1.9<br>3) 17.7 ± 1.9	The means of the 3 biological replicates were statistically analyzed. Friedman test followed by post-hoc multiple comparison analysis (Dunn).
2E	Transfection of cortical NPCs with the following constructs on DIV1:<br>1) kdcontrol<br>2) Pkd1-kd<br>3) Pkd2-kd<br>The cells were BrdU-labeled on DIV3 for three hours, fixed 24 h later on DIV4, and subjected to immunocytochemistry. The percentage of $BrdU^+$/$MAP2^+$ NPCs (NPCs differentiating to neurons after BrdU labeling) for each experimental condition was determined.	3	At least 600 transfected NPCs collected from 30 confocal images (10 per replicate) were analyzed for each experimental condition.	$BrdU^+$ $MAP2^+$ / $BrdU^+$ cells (%)<br><br>1) 20.2 ± 2.9<br>2) 12.0 ± 1.5<br>3) 13.8 ± 2.6	The means of the 3 biological replicates were statistically analyzed. Friedman test followed by post-hoc multiple comparison analysis (Dunn).
